# Supplementary material for: Two modes of evolution shape bacterial strain diversity in the mammalian gut for thousands of generations
Source: Nat Commun. 2022 Sep 24;13:5604. doi: 10.1038/s41467-022-33412-8 (PMC9509342; doi:10.1038/s41467-022-33412-8)
Supplement: Supplementary file 1 — Supplementary Information [file 41467_2022_33412_MOESM1_ESM.pdf]

Two modes of evolution shape bacterial strain diversity in the mammalian gut for thousands of generations

Frazão *et al*.

Supplementary Information

Supplementary Figures

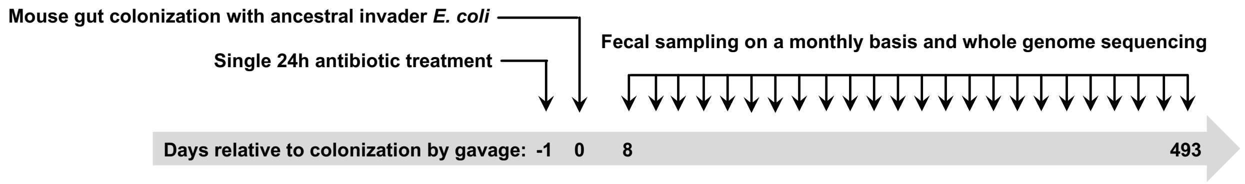

Supplementary Figure 1 | Experimental setup for *in vivo* Long-term Evolution Experiment (*in vivo* LTEE). Independently caged mice (n=9) were treated a single time during 24 hours with streptomycin (5 g/L) in drinking water. Afterwards the antibiotic was removed and colonization of the mice gut was performed by gavage with a suspension of ~10^8 colony forming units (CFUs) of streptomycin-resistant, yellow-fluorescent protein (YFP) *E. coli* ancestral invader clone. Fecal pellets were collected for 493 days post-gavage (~monthly basis) and stored in 15% glycerol at -80°C for later analysis.

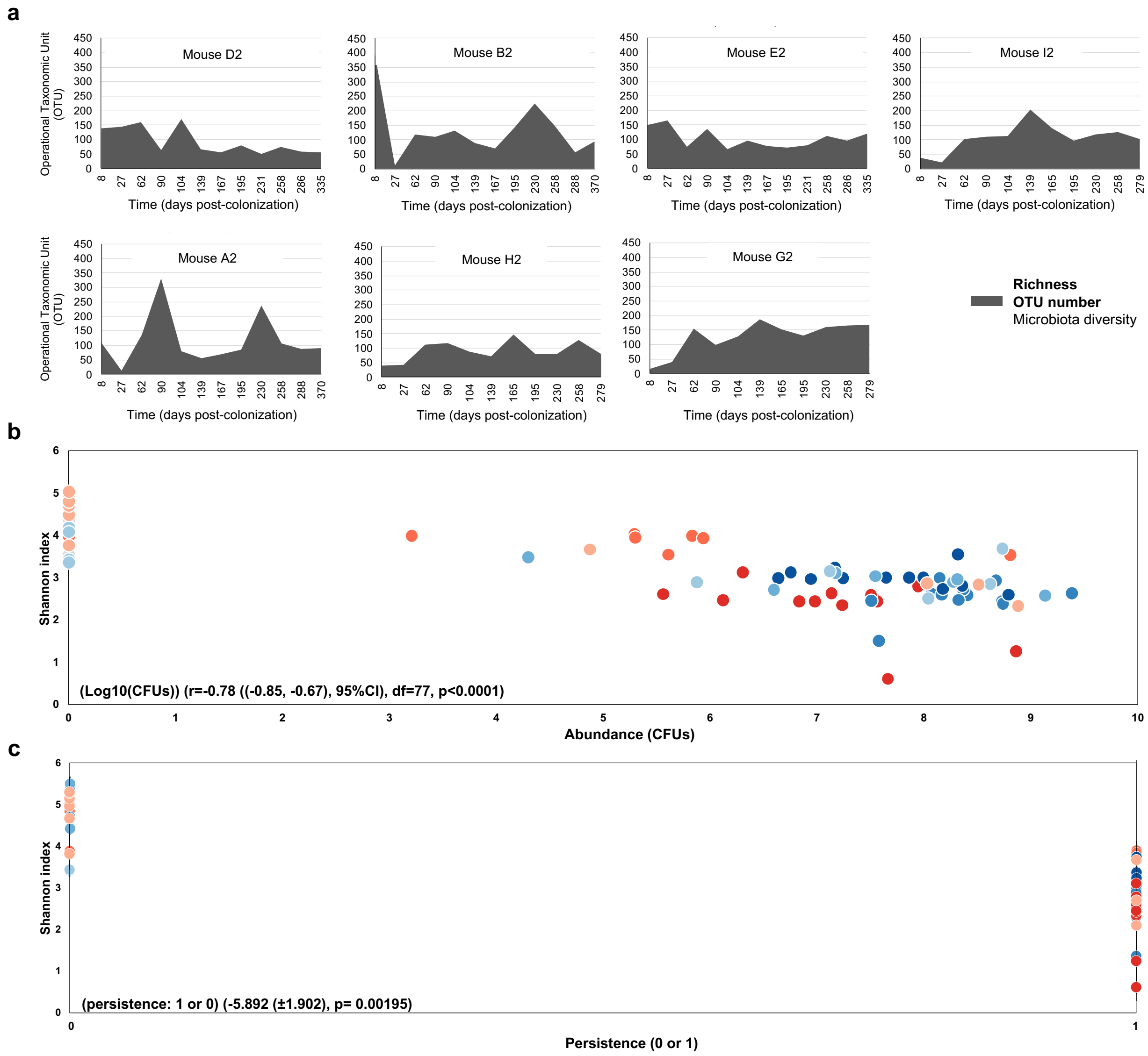

Supplementary Figure 2 | Microbiota diversity metrics. a, Richness of the microbiota in mice colonized with the invader *E. coli* (mice A2, B2, E2, I2, A2, H2 and G2). **b,** Negative correlation between the microbiota Shannon index and the invader *E. coli* abundance among the 9 mice of the experiment (Log10(CFUs)) (Repeated measures correlation, r=-0.78 ((-0.85, -0.67), 95%CI), df=77, p<0.0001) (Supplementary Data 2). **c,** Negative correlation between the microbiota Shannon index and the invader *E. coli* persistence among the 9 mice of the experiment (persistence: 1 or 0) (Linear Mixed-Effects Models, -5.892 (±1.902), p= 0.00195)

(Supplementary Data 2). Each colored circle represents a mouse. P-value was calculated by a repeated measures correlation.

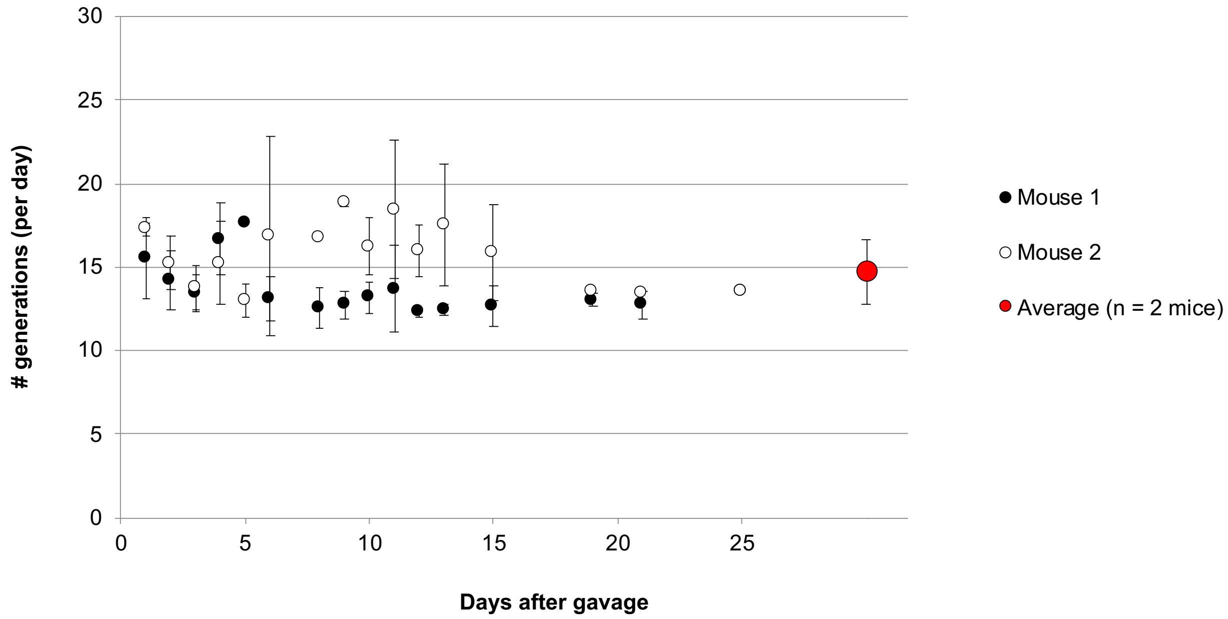

Supplementary Figure 3 | *E. coli* growth rate (generations per day) when colonizing the gut of mice treated with a single 24h streptomycin treatment (5g/L) before gavage. Ribosome content allowed us to assess the number of generations of the invader *E. coli* in the mouse gut (see Methods). Error bars represent Standard Deviation (SD) (Supplementary Data 4).

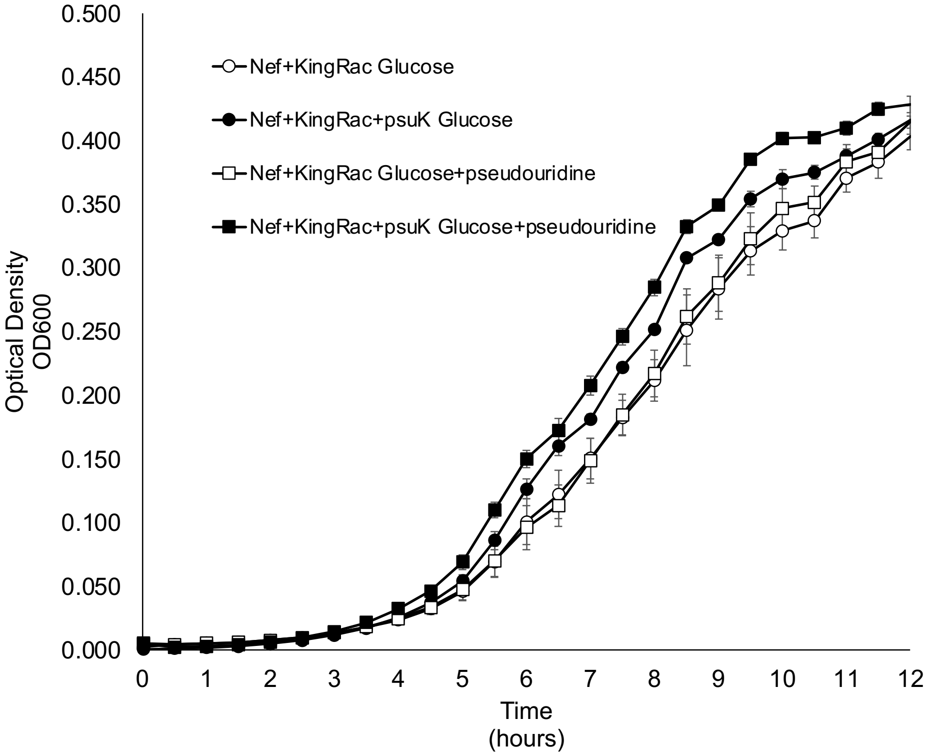

Supplementary Figure 4 | Growth curves of evolved clones. The growth of a double lysogen, carrying the prophages Nef and KingRac (Nef+KingRac), was compared to that of a clone carrying the two prophages and the *psuK*/*fruA* mutation (Nef+KingRac+*psuK*) *in vitro* (n=6 biological replicates per clone). Nef+KingRac+*psuK* has a higher maximum growth rate than Nef+KingRac (0.80 (0.04 SE) *vs.* 0.69 (0.02 SE), T-test P=0.00036) when grown in glucose (0.4%). When grown in glucose (0.4%) supplemented with pseudouridine (80□M), Nef+KingRac+*psuK* also has a significantly higher maximum growth rate than Nef+KingRac (0.79 (0.02 SE) *vs.* 0.63 (0.04 SE), a two-sided T-test P=0.0056). Each symbol (square and

circle) represents the mean value and error bars represent 2*Standard Error (2SE) (n = 3 biologically independent replicates). (Supplementary Data 17).

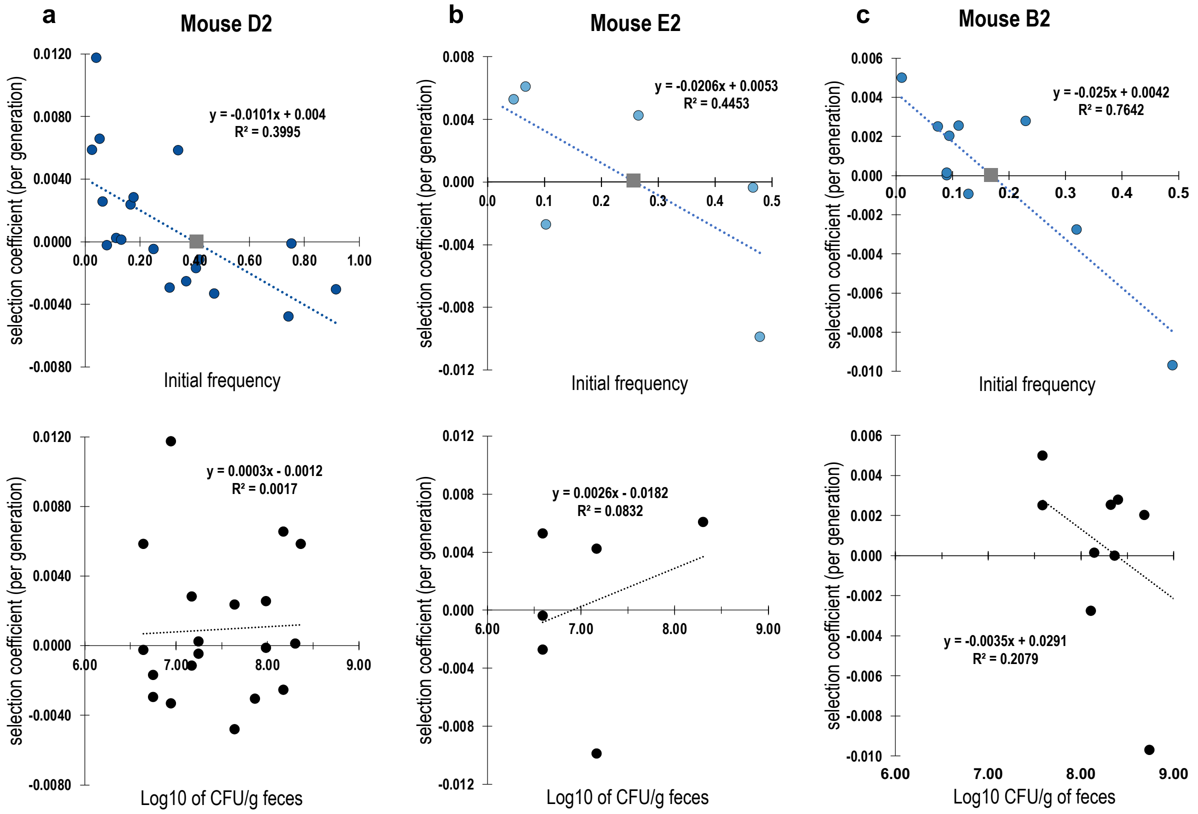

Supplementary Figure 5 | Evidence for negative frequency dependent selection in the frequency trajectory of *dgoR* mutations in mouse D2 and E2 and of *srlR* mutations in mouse B2. The change in the selection coefficient (*s* per generation), measured as the slope between Ln[frequency/(1-frequency)] of each allele at consecutive time points after the mutation was detected in the invader *E. coli* isolated from the feces of a mouse. A change in sign, from a positive *s* at lower frequencies to a negative *s* at higher frequencies, is a signal of negative-frequency dependent selection. Such form of selection should lead to a stable equilibrium frequency indicated by a grey square in the plots, if no other mutations would occur. We note that some forms of density dependent selection could also lead to correlations with initial frequency, however no correlation between *s* and population size (bottom panel), as measured from the CFUs/g of feces, was detected in mouse D2, and a very weak correlation

in mouse B2. **a,** In mouse D2, six different alleles were detected in *dgoR*. **b,** In mouse E2, four different alleles were detected in *dgoR*. **c,** In mouse B2, one single allele was detected in *srlR*. All alleles detected were used to calculate the correlations shown in the plots.

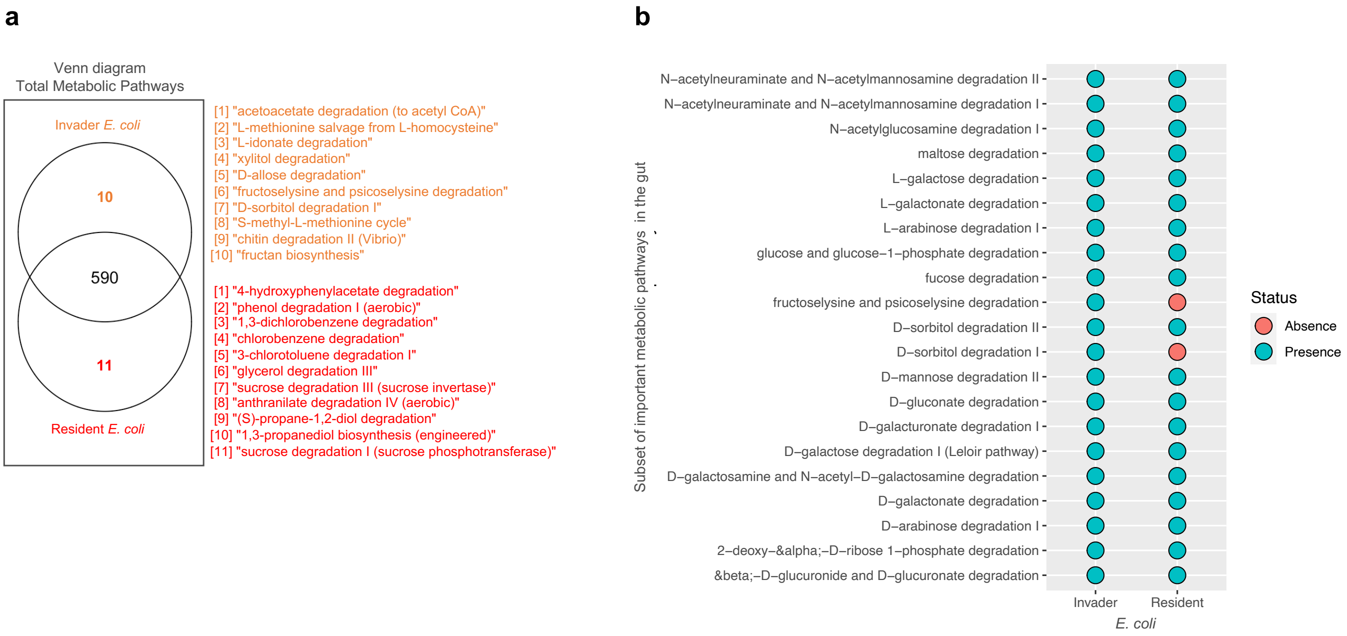

Supplementary Figure 6 | *E. coli* metabolic pathways. a, Venn diagram for the total metabolic pathways predicted for the Resident and Invader *E. coli* lineages. In orange and red are the unique pathways predicted for the Invader and Resident lineages, respectively. **b,** Comparison of a subset of the metabolic pathways important for *E. coli* colonization of the mouse intestine[1]. Red and green coloured circles represent the absence or presence of a metabolic pathway, respectively. Galactonate metabolic pathway is present both in the resident and invader *E. coli*. The resident lineage possesses a regulated *dgo* operon (galactonate operon) and competes for galactonate with the invader strain in the gut. If galactonate becomes inexistent in the gut, then the invader *dgoRKO* mutant, which constitutively spends energy to express the operon has fitness cost that the resident lineage does not have, since it has a regulated *dgo* operon. Prediction of the metabolic pathways was performed using the gapseq

tool[2]. Supplementary Data 15 presents all the predicted metabolic pathways from the genomes of resident and invader *E. coli* genomes.

References for Supplementary Figure 6

1. Fabich, A. J. et al. Comparison of Carbon Nutrition for Pathogenic and Commensal *Escherichia coli* Strains in the Mouse Intestine. Infect. Immun. **76**, 1143–1152 (2008).

2. Zimmermann, J., Kaleta, C. & Waschina, S. gapseq: informed prediction of bacterial metabolic pathways and reconstruction of accurate metabolic models. Genome Biol. **22**, 81 (2021).

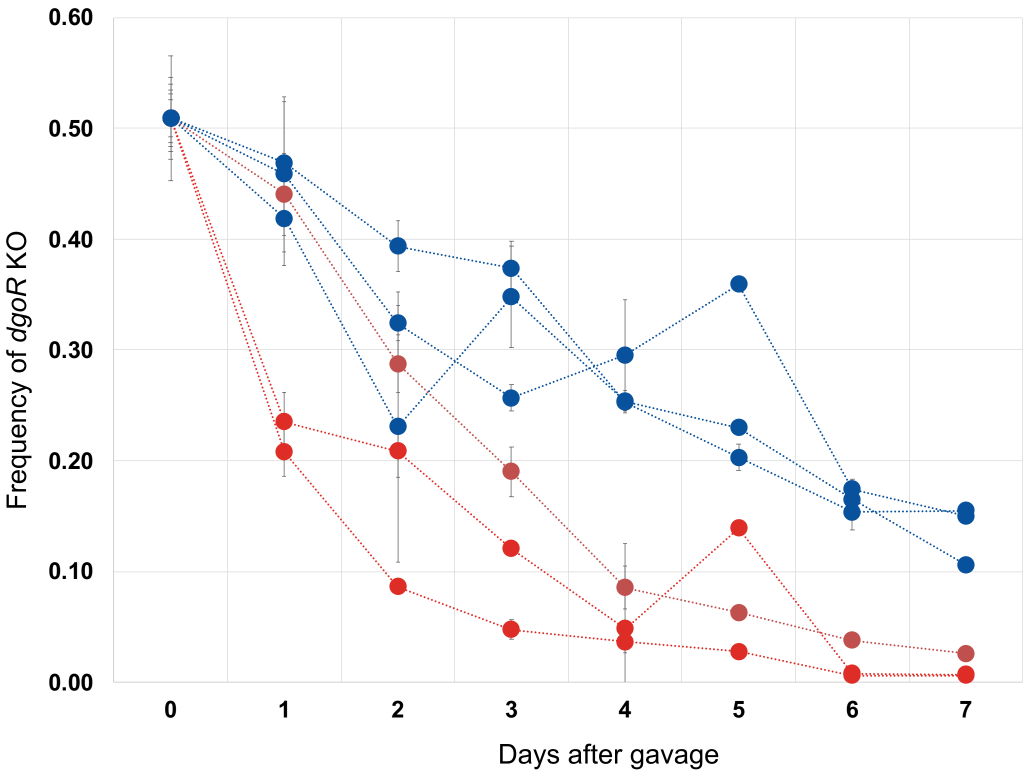

Supplementary Figure 7 | Frequency of the invader *E. coli dgoR* KO mutant when competing with ancestral in the mouse gut in the presence (red colour) or absence (blue colour) of the resident *E. coli* lineage. The presence of the resident (red colour) leads to a significant frequency decrease of the *dgoR* KO mutant, when compared with the resident's absence (blue colour). Linear mixed model followed by anova test: *dgoR* KO mutant frequency in presence (n = 3 biologically independent replicates) versus absence (n = 3 biologically independent replicates) of resident lineage, $P<0.0001$. Error bars represent SE (n = 3 technical replicates). Supplementary Data 18.

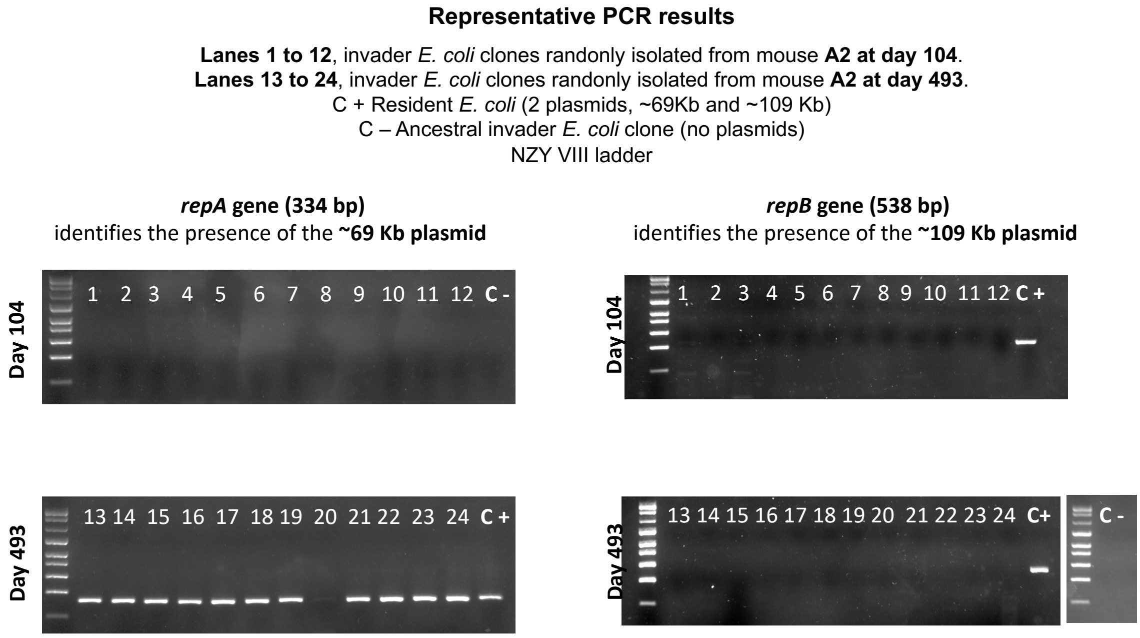

Supplementary Figure 8 | PCR-based plasmid frequency in the invader *E. coli* lineage isolated from the gut of mouse A2. The 334 bp portion of gene *repA,* that detects the ~69 Kb plasmid, inherited from the resident *E. coli* lineage was only detected at day 493 (7395 generations) being at 96% frequency (in 23 out of 24 clones isolated from mouse A2). The *repB* gene (identifying the ~109 Kb plasmid) was never detected in any of the clones isolated either at day 104 or 493. Positive and negative PCR reactions were repeated 3 times with identical results.

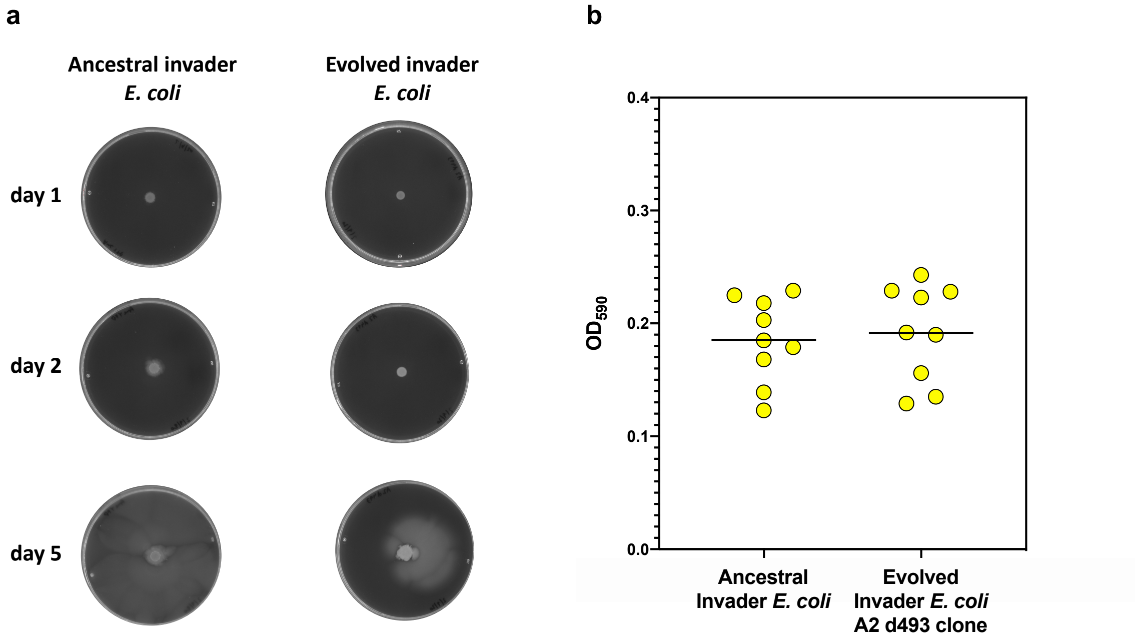

Supplementary Figure 9 | Gut adaptation appears to alter the motility while maintaining the biofilm capacity. a, Motility assays (ancestor and evolved clone isolated from mouse A2 at day 493). Each clone drop contained the same number of cells (OD600=0.1 – Bioscreen measurement). Images are representative of the observed phenotype. Three biological replicates were performed per clone. **b,** Biolfilm assay. Three biological replicates per clone, each with 3 technical replicates, were performed (Supplementary Data 24).

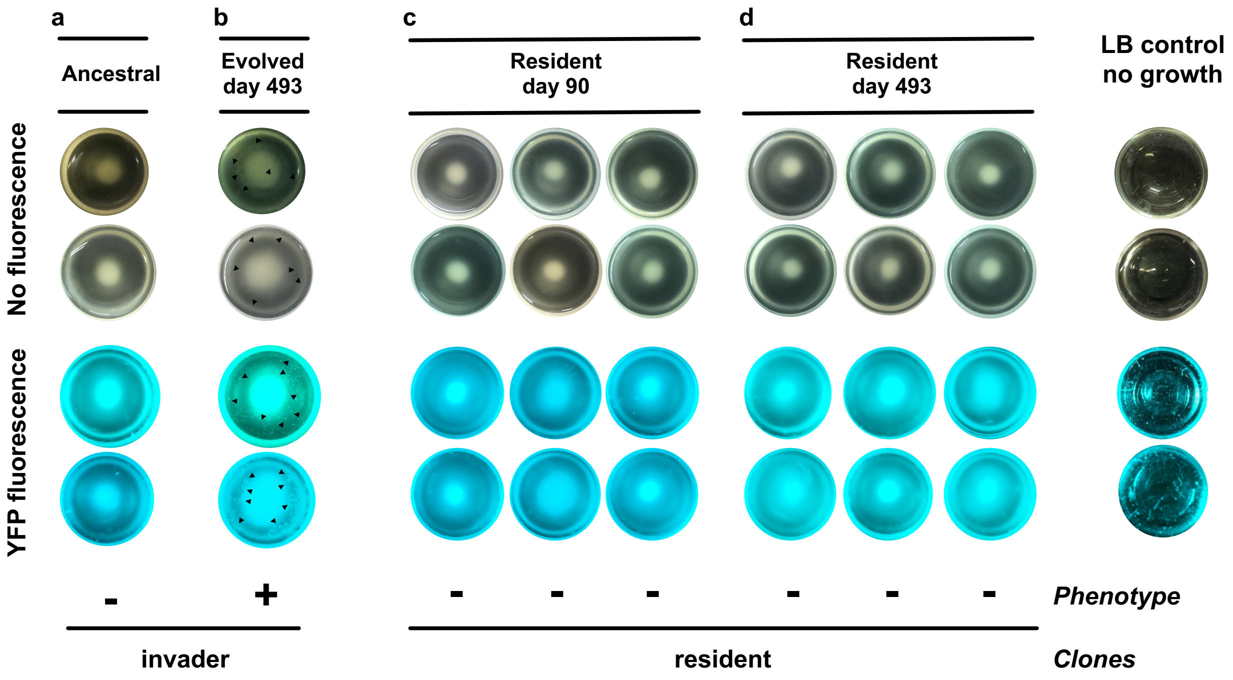

Supplementary Figure 10 | Evolved invader *E. coli* forms aggregates when growing in static conditions, while the resident did not present this trait. a, invader ancestral. **b,** invader evolved (day 493). **c,** resident (day 90). **d,** resident (day 493). Black arrow points to cell aggregates. Images are representative of the observed phenotype. Three biological replicates per clone, each with three technical replicates, were performed. Invader ancestral, and isolated from mouse A2 at day 493 express a Yellow Fluorescent Protein (YFP) marker. Blue/green LED transilluminator (FastGene) was used for verification of the YFP fluorescent of the invader and autofluorescence of the resident clones, which allow improved visualization of the phenotype.

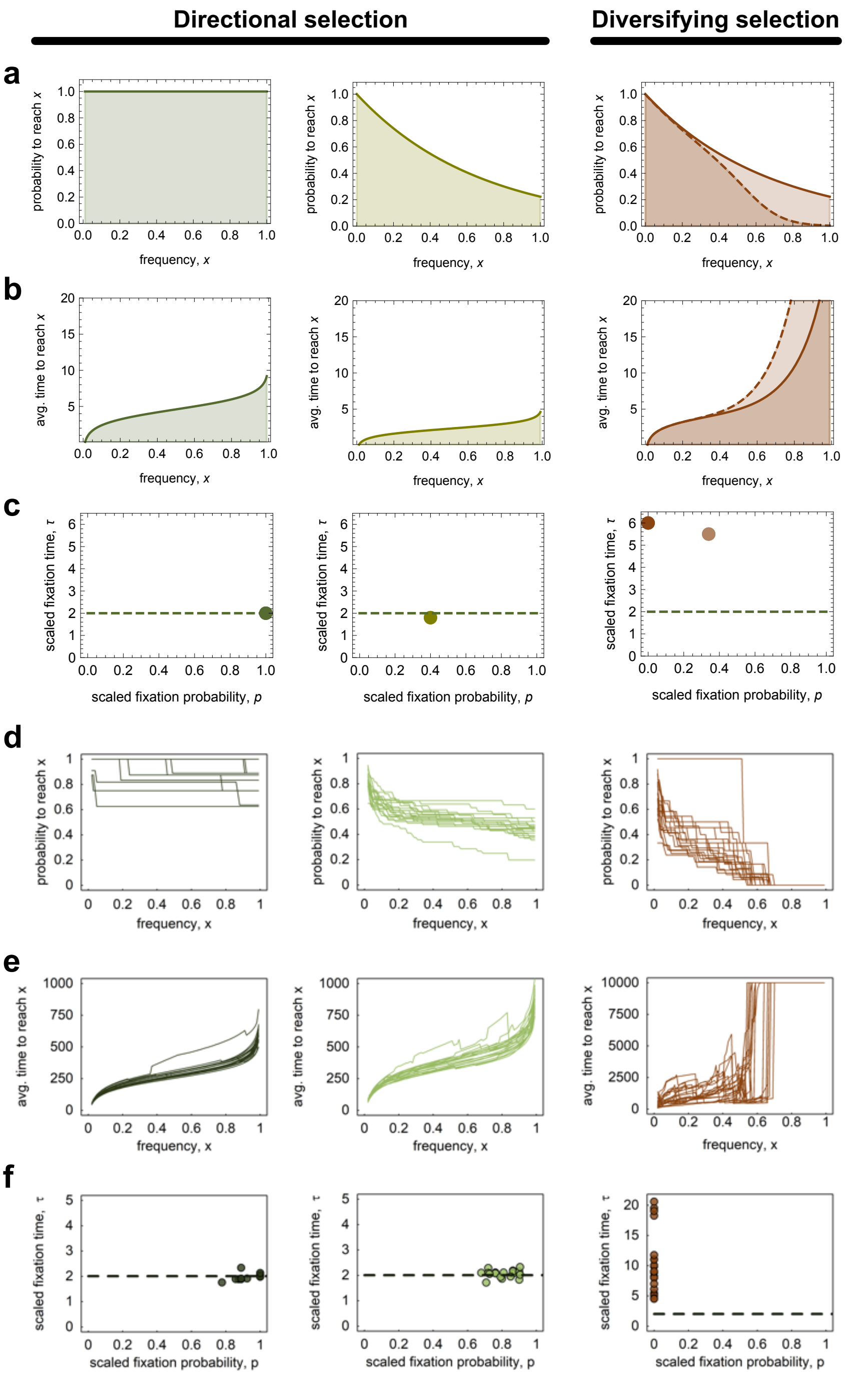

Supplementary Figure 11 | a-c, Frequency propagator, Coalescence time spectrum, p–τ summary statistics under different modes of adaptive evolution. Periodic sweeps under directional selection (left): high fixation probability and fast fixation of established, uniformly beneficial mutations. **Clonal interference under directional selection (center):** decreased

fixation probability, but even faster fixation of established, uniformly beneficial mutations. **Diversifying selection (right):** decreased fixation probability and slower fixation of established mutations with ecotype-specific fitness advantage. Fixations can be completely suppressed in case of strongly negative frequency-dependent selection across ecotypes (dashed lines); in that case, the total observation time of a mutation trajectory serves as a lower bound for the fixation time in the $p-\tau$ summary statistics. Times are measured in units of the inverse selection coefficient of individual sweeps. **d-f, Statistics of simulated adaptation under different mutation-selection models. Low-mutation regime under directional selection (left):** simulations of Wright-Fisher model with NU_b=0.01, exponentially-distributed selection effect with mean 0.05, where U_b is the rate of beneficial mutations per genome per generation. **High-mutation regime under directional selection (center):** simulations of Wright-Fisher model with NU_b=1, exponentially-distributed selection effect with mean 0.01. **Eco-evolutionary model of competition for resources (right):** simulations of the model from Amicone & Gordo[31], with NU=10, normally-distributed mutation effect with mean 0.05, 2 resources and initial fitness 0.9. All the simulations run for 10000 generations and have population size $N=10^7$. Lines and dots represent independent populations (n=20 replicate simulations of the same parameters).

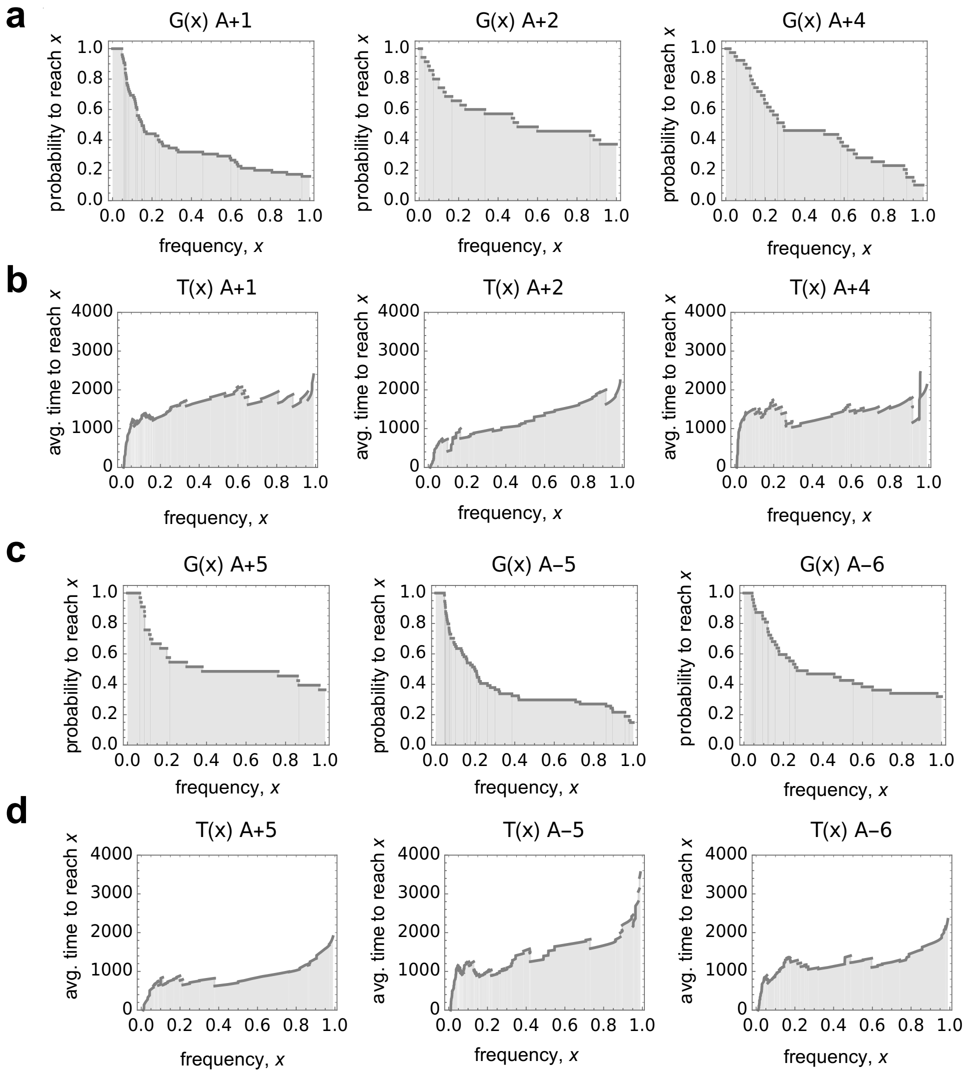

Supplementary Figure 12 | Statistics of *in-vitro* trajectories. Frequency propagators **(a, c)** and coalescence time spectrum **(b, d)** observed in the 6 Lenski populations that did not evolve a mutator phenotype over a period of 7500 generations (populations A+1, A+2, A+4, A+5, A-5 and A-6). Only mutations that reached at least 5% frequency where counted. Time is measured in generations. Under the conditions of the experiment 6.7 generations pass per day.

Uncropped Images of Supplementary Figure 8

The uncropped scan photos of the gels are supplied below.

***repA* gene (334 bp)**
identifies the presence of the **~69 Kb plasmid**

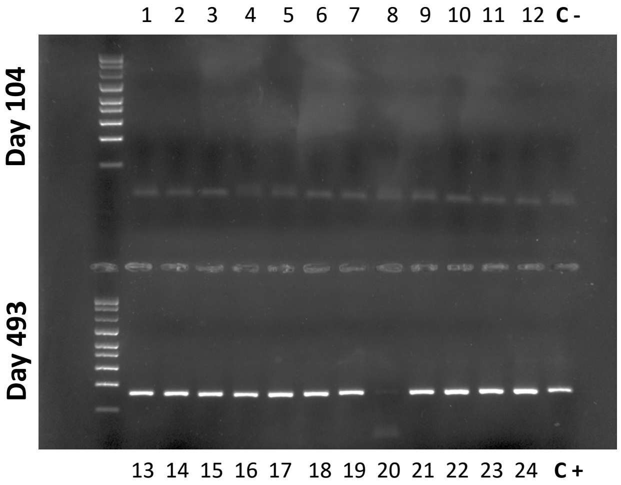

***repB* gene (538 bp)**
identifies the presence of the **~109 Kb plasmid**

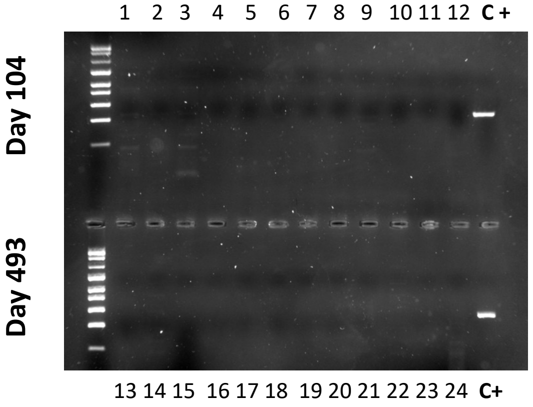

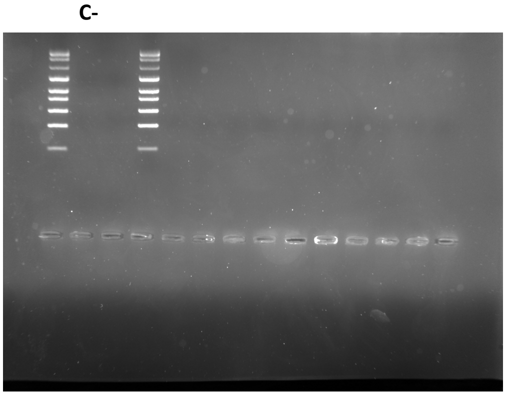

Code for Selection tests from the mutation frequency trajectories, in Mathematica version 12.2

```
(* Code for Frequency-Time Selection test *)
(* xmin=frequency threshold and s0=selection prior: doubling in 10 days *)
xmin = 0.01;s0=0.07;
(* a. Frequency propagator *)
(* Gu(x): number of trajectories reaching frequency x *)
Gu[x_, frq_]:= Sum[If[x<=Max[frq[[i]]],1,0],{i,1,Length[frq]}]

(* G(x): probability that a trajectory reaches frequency x *)
G[x_, frq_]:= Gu[x,frq]/Length[frq]

(* Gf(x): probability that a trajectory that has reached frequency x leads to
fixation *)
Gf[x_, frq_]:= G[1, frq]/G[x,frq]

(* b. Coalescence spectrum *)
(* log ratio transform, y = Log[x/(1-x)], of frequency trajectory *)
ylist[frql_, tl_] :=
(ymin = Log[xmin/(1 - xmin)];
imin = Position[frql, Select[frql, # > xmin &][[1]]][[1,1]];
(* regularized l.r. frequency trajectory *)
yl1= Table[{tl[[i]],Max[Log[Max[frql[[i]], 0.01]/(Max[1 - frql[[i]], 0.01])],
ymin]}, {i,imin,Length[tl]}];
ymax = Max[Transpose[yl1][[2]]];
(* yl: monotonic envelope *)
yl= Take[yl1,1];
If[Length[yl1]>1,
Do[If[Last[yl][[2]]<ymax,
yl=Append[yl, {yl1[[i,1]],Max[yl1[[i,2]],yl[[i-1,2]]+0.001]}],
Null],
{i,2,Length[yl1]}],
Null];
(* add inferred establishment time *)
ymin = Log[xmin/(1 - xmin)];
tmin= tl[[Position[tl,yl[[1,1]]][[1,1]]-1]];
s= If[Length[yl] > 1,
Max[(yl[[2,2]] - yl[[1,2]])/(yl[[2,1]]-yl[[1,1]]), s0],
s0];
yl= Prepend[yl, {Max[yl[[1,1]]-(yl[[1,2]]-ymin)/s, tmin],ymin}];
```

```
yl1= Prepend[yl1,yl[[1]]];
xl1 = Table[{yl1[[i,1]], 1/(1 + Exp[-yl1[[i,2]]])},{i,1,Length[yl1]}];
yl)

(* time to l.r. frequency y = Log[x/(1-x)] for a given trajectory *)
tint[y_,frql_,tl_]:=
( ylist[frql, tl];
tlist = Table[{yl[[k,2]], yl[[k,1]]- yl[[1,1]]}, {k,1,Length[yl]}];
If[y<=Last[tlist][[1]],Interpolation[tlist,InterpolationOrder->1][y], 0])

(* inverse time to l.r. frequency y = Log[x/(1-x)] for a given trajectory *)
tinv[y_,frql_,tl_]:=
( ylist[frql, tl];
tlist = Table[{yl[[k,2]], yl[[k,1]]- yl[[1,1]]}, {k,1,Length[yl]}];
If[y<=Last[tlist][[1]],1/Interpolation[tlist,InterpolationOrder->1][y], 0])

(* effective selection coefficient to l.r. frequency y = Log[x/(1-x)] for a given
trajectory *)
sint[y_,frql_,tl_]:=
( ylist[frql, tl];
tlist = Table[{yl[[k,2]], yl[[k,1]]- yl[[1,1]]}, {k,1,Length[yl]}];
If[y<=Last[tlist][[1]],(y-Log[0.01/0.99])/Interpolation[tlist,InterpolationOrder-
>1][y], 0])

(* average time to frequency x for a set of trajectories *)
tave[x_, frq_, tl_]:= Sum[tint[Log[x/(1-x)],frq[[j]],tl],
{j,1,Length[frq]}]/Gu[x,frq]

seff[x_, frq_, tl_]:= Sum[tinv[Log[x/(1-x)],frq[[j]],tl],
{j,1,Length[frq]}]/Gu[x,frq]

tas[x_,frq_, tl_,t0_]:=
(ymax = Max[Table[ylist[frq[[j]], tl];Last[yl][[2]],{j,1,Length[frq]}]];
xmax= 1/(1 + Exp[-ymax]);
If [x> xmax, t0,0])

(* Import data of mutational frequency trajectories *)
(* example for Mouse D2 sampling times (in days) *)
ttD= {0,8,27,62,90,104,139,167,195,231,258,286,335,436};
(* frequency data should be provided as a txt file where each raw has the
frequency of each mutation at each sampled time *)
```

```
frqD= Import["/TableofTimeFrequenciesMouseD2.txt", "Data"];
frqD=Map[(v=#;Map[If[#=="",0.0, #]&, v])&,frqD];
frqD = Table[Prepend[frqD[[j]],0], {j,1,Length[frqD]}];
frqD= Append[Drop[frqD,-1], Append[Last[frqD],0]];

(* Code for ploting Mutations trajectories shown in the top panels of Fig 2 and
Fig 3 *)
Show [ (* example for mouse D2*)
ListLinePlot[Table[ylist[Select[frqD,Max[#]<=0.95&][[j]], ttD]; xl1,{j,1,
Length[Select[frqD,Max[#]<=0.95&]]}],
Frame ->True,FrameTicks->{None,{0,0.5,1}}, PlotRange ->{0,1.05}, PlotStyle
-> Lighter[Gray]],ListLinePlot[Table[ylist[Select[frqD,Max[#]>fthers&][[j]],
ttD]; xl1,{j,1, Length[Select[frqD,Max[#]>0.95&]]}],
Frame ->True, PlotRange ->{0,1.05}, PlotStyle ->
RGBColor[8/255,81/255,156/255]],ImageSize->{500,50},AspectRatio-
>Full,LabelStyle->Directive[Black,FontFamily->"Arial"]]

(* Analysis of the mutation trajectories in Lenski non-mutator populations
evolved till 7500 generations,  only frequencies above 5% were considered *)
(* example for Ara-6 population*)
ttLam6=
{0,500,1500,2000,2500,3000,3500,4000,4500,5000,5500,6500,7000,7500};
frqLam6= Import["/Am6.txt", "Data"];
frqLam6=Map[(v=#;Map[If[#=="",0.0, #]&, v])&,frqLam6];
frqLam6 = Table[Prepend[frqLam6[[j]],0], {j,1,Length[frqLam6]}];
frqLam6= Append[Drop[frqLam6,-1], Append[Last[frqLam6],0]];

(* Plots of Figure 4A and 4B *)
asp=1.1;
Fig4ABmice=GraphicsGrid[{{
Plot[G[x,frqD],{x,0,1}, PlotRange ->{0,1.05}, PlotStyle ->
RGBColor["#8B4513"],LabelStyle->Directive[Black],
Filling -> Axis, Frame -> True,AspectRatio -> asp],
Plot[G[x,frqE],{x,0,1}, PlotRange ->{0,1.05},PlotStyle ->
RGBColor["#8B4513"], LabelStyle->Directive[Black],
Filling -> Axis,Frame -> True, AspectRatio -> asp],
Plot[G[x,frqB],{x,0,1}, PlotRange ->{0,1.05}, PlotStyle ->
RGBColor["#8B4513"],LabelStyle->Directive[Black],
Filling -> Axis, Frame -> True,AspectRatio -> asp],
Plot[G[x,frqI],{x,0,1}, PlotRange ->{0,1.05}, PlotStyle ->
RGBColor["#808000"],LabelStyle->Directive[Black],
```

```
Filling -> Axis, Frame -> True, AspectRatio -> asp],
Plot[G[x,frqG],{x,0,1}, PlotRange ->{0,1.05}, PlotStyle ->
RGBColor["#808000"],LabelStyle->Directive[Black],
Filling -> Axis,Frame -> True, AspectRatio -> asp],
Plot[G[x,frqA],{x,0,1}, PlotRange ->{0,1.05}, PlotStyle ->
RGBColor["#808000"],LabelStyle->Directive[Black],
Filling -> Axis, Frame -> True, AspectRatio -> asp],
Plot[G[x,frqH],{x,0,1}, PlotRange ->{0,1.05}, PlotStyle ->
RGBColor["#556B2F"],LabelStyle->Directive[Black],
Filling -> Axis, Frame -> True, AspectRatio -> asp]
},
{Plot[{tave[x, frqD, ttD],tas[x,frqD, ttD,436] }, {x,0.01,0.99}, PlotRange ->
{{0,1},{0,450}},
PlotStyle ->  RGBColor["#8B4513"],LabelStyle->Directive[Black],Frame ->
True,Filling -> Axis, AspectRatio -> asp],
Plot[tave[x, frqE, ttE], {x,0.01,0.99}, PlotRange -> {0,165},
PlotStyle -> RGBColor["#8B4513"],LabelStyle->Directive[Black],Frame ->
True,Filling -> Axis, AspectRatio -> asp],
Plot[tave[x, frqB, ttB], {x,0.01,0.99}, PlotRange -> {0,450},
PlotStyle -> RGBColor["#8B4513"],LabelStyle->Directive[Black],Frame ->
True,Filling -> Axis,  AspectRatio -> asp],
Plot[tave[x, frqI, ttI], {x,0.01,0.99}, PlotRange -> {0,165},
PlotStyle -> RGBColor["#808000"],LabelStyle->Directive[Black],Frame ->
True,Filling -> Axis, AspectRatio -> asp],
Plot[tave[x, frqG, ttG], {x,0.01,0.99}, PlotRange -> {0,165},
PlotStyle -> RGBColor["#808000"],LabelStyle->Directive[Black],Frame ->
True,Filling -> Axis, AspectRatio -> asp],
Plot[tave[x, frqA, ttA], {x,0.01,0.99}, PlotRange -> {0,165},
PlotStyle -> RGBColor["#808000"],LabelStyle->Directive[Black],Frame ->
True,Filling -> Axis, AspectRatio -> asp],
Plot[tave[x, frqH, ttH], {x,0.01,0.99}, PlotRange -> {0,165},
PlotStyle -> RGBColor["#556B2F"],LabelStyle->Directive[Black],Frame ->
True,Filling -> Axis, AspectRatio -> asp]
}},ImageSize->1100]

(* Code for Ploting Fig 4C *)
fG1=0.95;fG2=0.3;
Fig4C=Show[ListPlot[{
{G[fG1,frqD]/G[fG2,frqD],282/(tave[fG2,frqD,ttD])}}, PlotStyle-> {PointSize-
>0.07, RGBColor["#8B4513"]},
```

```
PlotRange -> {{-0.04,1.04},{0,5.5}}, Frame -> True, FrameLabel-> {"scaled fixation probability, p", "scaled fixation time, \[Tau]"},LabelStyle->Directive[Black,FontFamily->"Arial"]],
ListPlot[{{G[fG1,frqE]/G[fG2,frqE],tave[fG1,frqE,ttE]/tave[fG2,frqE,ttE]}},PlotStyle->{PointSize->0.07, RGBColor["#8B4513"]}],
ListPlot[{{G[fG1,frqB]/G[fG2,frqB],tave[fG1,frqB,ttB]/tave[fG2,frqB,ttB]}},PlotStyle->{PointSize->0.07, RGBColor["#8B4513"]}],
ListPlot[{{G[fG1,frqI]/G[fG2,frqI],tave[fG1,frqI,ttI]/tave[fG2,frqI,ttI]}},PlotStyle->{PointSize->0.055,RGBColor["#808000"]}],
ListPlot[{{G[fG1,frqA]/G[fG2,frqA],tave[fG1,frqA,ttA]/tave[fG2,frqA,ttA]}},
PlotStyle->{PointSize->0.07,RGBColor["#808000"]}],
ListPlot[{{G[fG1,frqH]/G[fG2,frqH],tave[fG1,frqH,ttH]/tave[fG2,frqH,ttH]}},
PlotStyle->{PointSize->0.07,RGBColor["#556B2F"]}],
ListPlot[{{G[fG1,frqG]/G[fG2,frqG],tave[fG1,frqG,ttG]/tave[fG2,frqG,ttG]}},
PlotStyle->{PointSize->0.055,RGBColor["#808000"]}],
ListPlot[{
{G[fG1,frqLam5]/G[fG2,frqLam5],tave[fG1,frqLam5,ttLam5]/tave[fG2,frqLam5,ttLam5]},
{G[fG1,frqLam6]/G[fG2,frqLam6],tave[fG1,frqLam6,ttLam6]/tave[fG2,frqLam6,ttLam6]},
{G[fG1,frqLap1]/G[fG2,frqLap1],tave[fG1,frqLap1,ttLap1]/tave[fG2,frqLap1,ttLap1]},
{G[fG1,frqLap2]/G[fG2,frqLap2],tave[fG1,frqLap2,ttLap2]/tave[fG2,frqLap2,ttLap2]},
{G[fG1,frqLap4]/G[fG2,frqLap4],tave[fG1,frqLap4,ttLap4]/tave[fG2,frqLap4,ttLap4]},
{G[fG1,frqLap5]/G[fG2,frqLap5],tave[fG1,frqLap5,ttLap5]/tave[fG2,frqLap5,ttLap5]}},PlotMarkers->"OpenMarkers",PlotStyle->{PointSize->0.07,RGBColor["#808000"]}],
Plot[2,{x,0,1}, PlotStyle ->{RGBColor["#556B2F"],Dashed}], ImageSize->{152,182},AspectRatio->Full]

(* Figure S10 Cartoons of test statistics for different types of selection *)
aspect=0.75;
figS10teoretical=GraphicsGrid[{{
Plot[1,{x,0.01,1}, PlotRange ->{0,1.09}, PlotStyle ->
RGBColor["#8B0000"],LabelStyle->Directive[Black],
Filling -> Axis, Frame -> True, FrameLabel->{"frequency, x", "probability to reach x"}, AspectRatio -> aspect],
Plot[Exp[-1.5x],{x,0,1}, PlotRange ->{0,1.09},PlotStyle ->
RGBColor["#DC143C"], LabelStyle->Directive[Black],
```

```
Filling -> Axis, Frame -> True, FrameLabel->{"frequency, x", "probability to
reach x"},AspectRatio -> aspect],
Plot[{Exp[-1.5 x], Exp[-1.5x] /(1+Exp[10(x-0.6)])},{x,0,1}, PlotRange -
>{0,1.09},
PlotStyle -> {{RGBColor["#000080"]},{Dashed,
RGBColor["#000080"]}},LabelStyle->Directive[Black],
Filling -> Axis, Frame -> True, FrameLabel->{"frequency, x", "probability to
reach x"},AspectRatio -> aspect]
},
{Plot[(Log[x/(1-x)]- Log[0.01/0.99]), {x,0.01,0.99}, PlotRange -> {0.1,20},
PlotStyle -> RGBColor["#8B0000"],LabelStyle->Directive[Black],
Frame -> True,Filling -> Axis,FrameLabel -> {"frequency, x", "avg. time to
reach x"}, AspectRatio -> aspect],
Plot[(Log[x/(1-x)]- Log[0.01/0.99])/2, {x,0.01,0.99}, PlotRange -> {0.1,20},
PlotStyle -> RGBColor["#DC143C"],LabelStyle->Directive[Black],Frame ->
True,Filling -> Axis, FrameLabel -> {"frequency, x", "avg. time to reach x"},
AspectRatio -> aspect],
Plot[ {(Log[x/(1-x)]- Log[0.01/0.99]) +12 x(Exp[x^4]-1),(Log[x/(1-x)]-
Log[0.01/0.99]) +40 x(Exp[x^4]-1)}, {x,0.01,0.99}, PlotRange -> {0.1,20},
PlotStyle -> {{RGBColor["#000080"]},{Dashed,
RGBColor["#000080"]}},LabelStyle->Directive[Black],
Frame -> True,Filling -> Axis,FrameLabel -> {"frequency, x", "avg. time to
reach x"}, AspectRatio -> aspect]}}, ImageSize -> 600]

FigSup10Bottom1=Show[ListPlot[{{1,2}}, PlotStyle-> {PointSize->0.07,
RGBColor["#DC143C"]},
PlotRange -> {{-0.04,1.04},{0,6.5}}, Frame -> True, FrameLabel-> {"scaled
fixation probability, p", "scaled fixation time, \[Tau]"},LabelStyle-
>Directive[Black,FontFamily->"Arial"]],
Plot[2,{x,0,1}, PlotStyle ->{RGBColor["#DC143C"],Dashed}], ImageSize-
>{152,182},AspectRatio->aspect]

FigSup10Bottom2=Show[ListPlot[{{0.4,1.8}}, PlotStyle-> {PointSize->0.07,
RGBColor["#DC143C"]},
PlotRange -> {{-0.04,1.04},{0,6.5}}, Frame -> True, FrameLabel-> {"scaled
fixation probability, p", "scaled fixation time, \[Tau]"},LabelStyle-
>Directive[Black,FontFamily->"Arial"]],
Plot[2,{x,0,1}, PlotStyle ->{RGBColor["#DC143C"],Dashed}], ImageSize-
>{152,182},AspectRatio->aspect]
```

```
FigSup10Bottom3=Show[ListPlot[{{0,6}}, PlotStyle-> {PointSize->0.07,
RGBColor["#000080"]},
PlotRange -> {{-0.04,1.04},{0,6.5}}, Frame -> True, FrameLabel-> {"scaled
fixation probability, p", "scaled fixation time, \[Tau]"},LabelStyle-
>Directive[Black,FontFamily->"Arial"]],
ListPlot[{{0.34,5.5}},PlotStyle->{PointSize-
>0.07,Lighter[RGBColor["#000080"]]}],
Plot[2,{x,0,1}, PlotStyle ->{RGBColor["#DC143C"],Dashed}], ImageSize-
>{152,182},AspectRatio->aspect]
```
