# Supplementary material for: Two modes of evolution shape bacterial strain diversity in the mammalian gut for thousands of generations
Source: Nat Commun. 2022 Sep 24;13:5604. doi: 10.1038/s41467-022-33412-8 (PMC9509342; doi:10.1038/s41467-022-33412-8)
Supplement: Supplementary file 3 — Description of Additional Supplementary Files [file 41467_2022_33412_MOESM3_ESM.docx]

Two modes of evolution shape bacterial strain diversity in the mammalian gut for thousands of generations

Frazão *et al*.

Supplementary Data Descriptive Legends

**Supplementary Data 1.** *Escherichia coli* (*E. coli*) loads (CFU per gram of feces)

**Supplementary Data 2.** Microbiota diversity along time

**Supplementary Data 3.** Abundance of the Operational Taxonomic Units (OTU) at the phylum level (level 2) within the mice gut microbiota along time

**Supplementary Data 4.** *E. coli* growth rate (generations per day) when colonizing the gut of mice treated with a single 24h streptomycin treatment (5g/L) before gavage

**Supplementary Data 5.** Rates of molecular evolution

**Supplementary Data 6.** *E. coli* mutation dynamics in the mouse gut and *in vitro*

**Supplementary Data 7.** Mutational parallelism for adaptation to the mouse gut and assessment of negative Frequency Dependent Selection (negative-FDS)

**Supplementary Data 8.** Evolutionary changes detected in the invader *E. coli* lineage population isolated from mouse A2

**Supplementary Data 9.** Evolutionary changes detected in the invader *E. coli* lineage population isolated from mouse B2

**Supplementary Data 10.** Evolutionary changes detected in the invader *E. coli* lineage population isolated from mouse D2

**Supplementary Data 11.** Evolutionary changes detected in the invader *E. coli* lineage population isolated from mouse E2

**Supplementary Data 12.** Evolutionary changes detected in the invader *E. coli* lineage population isolated from mouse G2

**Supplementary Data 13.** Evolutionary changes detected in the invader *E. coli* lineage population isolated from mouse H2

**Supplementary Data 14.** Evolutionary changes detected in the invader *E. coli* lineage population isolated from mouse I2

**Supplementary Data 15.** *E. coli* predicted metabolic pathways for the Resident and Invader lineages using the gapseq tool

**Supplementary Data 16.** Differential gene expression levels of the invader evolved clones compared with the invader ancestral clone

**Supplementary Data 17.** Growth curves of evolved clones (OD600 values)

**Supplementary Data 18.** Competition (1:1) between invader *E. coli* dgoR KO mutant and the ancestral clone, in the presence or absence of the resident *E. coli* lineage - loads (CFU per gram of feces)

**Supplementary Data 19.** PCR-based plasmid frequency in the invader *E. coli* lineage isolated from the gut of mouse A2

**Supplementary Data 20.** Induction rate (per hour) of invader and resident *E. coli* clones isolated from the gut of mice

**Supplementary Data 21.** Evolutionary changes detected in the resident *E. coli* lineage population isolated from mouse A2 and maximum frequency of genes targeted by de novo mutation (invader and resident *E. coli*)

**Supplementary Data 22.** Gene hits between resident and invader *E. coli* clones

**Supplementary Data 23.** Evolutionary changes detected in invader *E. coli* clones isolated from the mouse gut

**Supplementary Data 24.** Quantitative biofilm assay

**Supplementary Data 25.** Competition in the gut of new mice (female and male) between invader evolved *E. coli* populations and the ancestral clone - loads (CFU per gram of feces)

**Supplementary Data 26.** Growth rate (per hour) of invader evolved *E. coli* isolated from the mouse gut
