# Supplementary material for: Two modes of evolution shape bacterial strain diversity in the mammalian gut for thousands of generations
Source: Nat Commun. 2022 Sep 24;13:5604. doi: 10.1038/s41467-022-33412-8 (PMC9509342; doi:10.1038/s41467-022-33412-8)
Supplement: Supplementary file 5 — Reporting Summary [file 41467_2022_33412_MOESM5_ESM.pdf]

nature portfolio

Corresponding author(s): Nelson Frazão and Isabel Gordo

Last updated by author(s): Setember 7, 2022

Reporting Summary

Nature Portfolio wishes to improve the reproducibility of the work that we publish. This form provides structure for consistency and transparency in reporting. For further information on Nature Portfolio policies, see our Editorial Policies and the Editorial Policy Checklist.

Statistics

For all statistical analyses, confirm that the following items are present in the figure legend, table legend, main text, or Methods section.

n/a	Confirmed	
☐	☒	The exact sample size (*n*) for each experimental group/condition, given as a discrete number and unit of measurement
☐	☒	A statement on whether measurements were taken from distinct samples or whether the same sample was measured repeatedly
☐	☒	The statistical test(s) used AND whether they are one- or two-sided<br>*Only common tests should be described solely by name; describe more complex techniques in the Methods section.*
☐	☒	A description of all covariates tested
☐	☒	A description of any assumptions or corrections, such as tests of normality and adjustment for multiple comparisons
☐	☒	A full description of the statistical parameters including central tendency (e.g. means) or other basic estimates (e.g. regression coefficient) AND variation (e.g. standard deviation) or associated estimates of uncertainty (e.g. confidence intervals)
☐	☒	For null hypothesis testing, the test statistic (e.g. *F*, *t*, *r*) with confidence intervals, effect sizes, degrees of freedom and *P* value noted<br>*Give P values as exact values whenever suitable.*
☒	☐	For Bayesian analysis, information on the choice of priors and Markov chain Monte Carlo settings
☒	☐	For hierarchical and complex designs, identification of the appropriate level for tests and full reporting of outcomes
☐	☒	Estimates of effect sizes (e.g. Cohen's *d*, Pearson's *r*), indicating how they were calculated

Our web collection on statistics for biologists contains articles on many of the points above.

Software and code

Policy information about availability of computer code

Data collection	R version 3.6.1 was used to generate simulation data.
Data analysis	Software used for whole-genome sequencing and analysis: DADA2 version 1.14, fastp version 0.20.0, BWA-sampe version 0.7.17, MOSAIK version 2.7, Breseq version 0.35.1, samtools version 1.9, freebayes version 0.9.21, ismapper version 2, panISa version 0.1.6, IGV version 2.7, and BBsplit (part of BBMap version 38.9).<br><br>Statistics and Reproducibility: Correlation between microbiota diversity measures and E. coli loads (CFU) or persistence (1-presence or 0-absence) was performed in R using the statistical package rmcorr (version 0.5.2)56 and lme4 (version 1.1-10)57, respectively. The rate of accumulation of new ISs in vivo was compared using Wilcoxon paired signed ranked test for expected and observed insertions, while the rate of selective sweeps correlation was performed using the Spearman Correlation test. Selective sweeps were taken to be mutations or HGT events that reached >95% frequency in the population and kept high frequency until the end of the colonization. Statistical analysis of prophage induction as well as biofilm levels was performed using the Mann-Whitney test in GraphPad Prism (version 8.4.3). A single sample T-Test was used test if the growth rate of evolved invader clones deviates from the mean of the ancestral. A Wilcoxon rank sum test with continuity correction was used to compare the relative expression levels of the evolved clones with the ancestral. P values of <0.05 were considered significant.<br>Pearson correlation tests between the frequency and the change in frequency of a mutation were performed to search for evidence of negative frequency dependent selection. These were conducted for every mutation that showed parallelism and for each mouse, provided that the mutation was detected in at least four time points. The correlations were calculated in R with cor.test, used for association between paired samples.<br>Linear mixed models (R package nlme, v3.158) were used to analyze the temporal dynamics of the dgoR KO mutant frequency in the presence or absence of the resident E. coli. The frequency of the dgoR KO mutant was log10 transformed to meet the assumptions of parametric

statistics.
Sample size in animal experiments was chosen according to institutional directives and in accordance with the guiding principles underpinning humane use of animals in scientific research. No data was excluded from the analysis. The experiments were randomized with animals being assigned arbitrarily to each experiment. The investigators were not blind towards the animal experiments.

Code Availability: The code for the Frequency-time statistics of mutation trajectories is available in the Methods. The code for the simulations is available at: https://github.com/AmiconeM/2EvolutionModes and is linked to Zenodo database: https://doi.org/10.5281/zenodo.7043896 (doi: 10.5281/zenodo.7043896).

For manuscripts utilizing custom algorithms or software that are central to the research but not yet described in published literature, software must be made available to editors and reviewers. We strongly encourage code deposition in a community repository (e.g. GitHub). See the Nature Portfolio guidelines for submitting code & software for further information.

Data

Policy information about availability of data

All manuscripts must include a data availability statement. This statement should provide the following information, where applicable:

- Accession codes, unique identifiers, or web links for publicly available datasets
- A description of any restrictions on data availability
- For clinical datasets or third party data, please ensure that the statement adheres to our policy

Data Availability The raw population sequencing and 16s rDNA data generated in this study have been deposited in the open access sequence read archive (part of the National Center for Biotechnology Information) under accession code (Bioproject) PRJNA666769 [https://www.ncbi.nlm.nih.gov/bioproject/?term=PRJNA666769]. This data is neither restricted nor protected due to data privacy laws. Reference genomes were used for the alignment of invader (K-12 (substrain MG1655; Accession Number: NC_000913.2)) and resident (Accession Number: SAMN15163749) E. coli genomes. The Greengenes database (http://greengenes.lbl.gov) was used for microbiota taxonomic analysis.

Human research participants

Policy information about studies involving human research participants and Sex and Gender in Research.

Reporting on sex and gender	Not applicable.
Population characteristics	Not applicable.
Recruitment	Not applicable.
Ethics oversight	Not applicable.

Note that full information on the approval of the study protocol must also be provided in the manuscript.

Field-specific reporting

Please select the one below that is the best fit for your research. If you are not sure, read the appropriate sections before making your selection.

[x] Life sciences [ ] Behavioural & social sciences [ ] Ecological, evolutionary & environmental sciences

For a reference copy of the document with all sections, see nature.com/documents/nr-reporting-summary-flat.pdf

Life sciences study design

All studies must disclose on these points even when the disclosure is negative.

Sample size	The sample size used to study in vivo evolution required no specific calculations. Sample size was chosen according to institutional directives and in accordance with the guiding principles underpinning humane use of animals in scientific research. Evolution experiments in the gut of C57BL/6J mice were performed independently with a sample size of n = 7. Competition experiments in the gut of C57BL/6J mice were performed using a sample size of n = 4 (for each relative fitness experiment) and n = 3 (for each dgoR experiment).
Data exclusions	No data was excluded.
Replication	In the present study the in vivo evolution experiments were replicated independently in different mice caged individually (n = 7). Seven mice (A2, B2, D2, E2, G2, H2 and I2) were successfully colonized with the invader E. coli, while two mice (C2 and F2) failed to be colonized. Other experiments requiring controls (e.g. plasmid presence, prophage induction or growth rates) showed similar results every time they were replicated.

Randomization	Not relevant for this study, as the genetic background of the mice is the same and all received antibiotic treatment.
Blinding	The investigators were not blind towards the animal experiments. The experimental evolution involved a temporal series of data where key observations (e.g. rate of adaptive molecular evolution) are based on simple quantitative measurements (e.g. sum of allele frequencies), which are not bound to be biased by the investigator.

Reporting for specific materials, systems and methods

We require information from authors about some types of materials, experimental systems and methods used in many studies. Here, indicate whether each material, system or method listed is relevant to your study. If you are not sure if a list item applies to your research, read the appropriate section before selecting a response.

Materials & experimental systems

n/a	Involved in the study	
☒	☐	Antibodies
☒	☐	Eukaryotic cell lines
☒	☐	Palaeontology and archaeology
☐	☒	Animals and other organisms
☒	☐	Clinical data
☒	☐	Dual use research of concern

Methods

n/a	Involved in the study	
☒	☐	ChIP-seq
☒	☐	Flow cytometry
☒	☐	MRI-based neuroimaging

Animals and other research organisms

Policy information about studies involving animals; ARRIVE guidelines recommended for reporting animal research, and Sex and Gender in Research

Laboratory animals	All mice (Mus musculus) used in this study were supplied by the Rodent Facility at Instituto Gulbenkian de Ciência (IGC) and were given ad libitum access to food (Rat and Mouse No.3 Breeding (Special Diets Services) and water. Mice (6-8 weeks old) were kept in individually ventilated cages under specified pathogen free (SPF) barrier conditions at the IGC animal facility. The room where the mice were kept was at 20-24°C and 40-60% humidity with a 12-hour light-dark cycle.
Wild animals	The study did not involve wild animals.
Reporting on sex	The present study used mostly female animals, this is why we do not report sex-based analysis. Seven females were used for the long-term evolution experiment and six for the dgoR competition. In the competition assays, both male (n = 8) and female (n = 8) mice were used.
Field-collected samples	The study did not involve field-collected samples.
Ethics oversight	This research project was ethically reviewed and approved by the Ethics Committee of the Instituto Gulbenkian de Ciência (license reference: A009.2018), and by the Portuguese National Entity that regulates the use of laboratory animals (DGAV - Direção Geral de Alimentação e Veterinária (license reference: 008958). All experiments conducted on animals followed the Portuguese (Decreto-Lei nº 113/2013) and European (Directive 2010/63/EU) legislations, concerning housing, husbandry and animal welfare.

Note that full information on the approval of the study protocol must also be provided in the manuscript.
